# Supplementary material for: The Epithelial-Mesenchymal Transcription Factor Slug Predicts Survival Benefit of Up-Front Surgery in Head and Neck Cancer
Source: Cancers (Basel). 2021 Feb 12;13(4):772. doi: 10.3390/cancers13040772 (PMC7918715; doi:10.3390/cancers13040772)
Supplement: Supplementary file 1 [file cancers-13-00772-s001.pdf]

cancers

MDPI

Supplementary Materials: The Epithelial-Mesenchymal Transcription Factor Slug Predicts Survival Benefit of up-front Surgery in Head and Neck Cancer

Herbert Riechelmann, Teresa Bernadette Steinbichler, Susanne Sprung, Matthias Santer, Annette Runge, Ute Ganswindt, Gabriele Gamerith and Jozsef Dudas

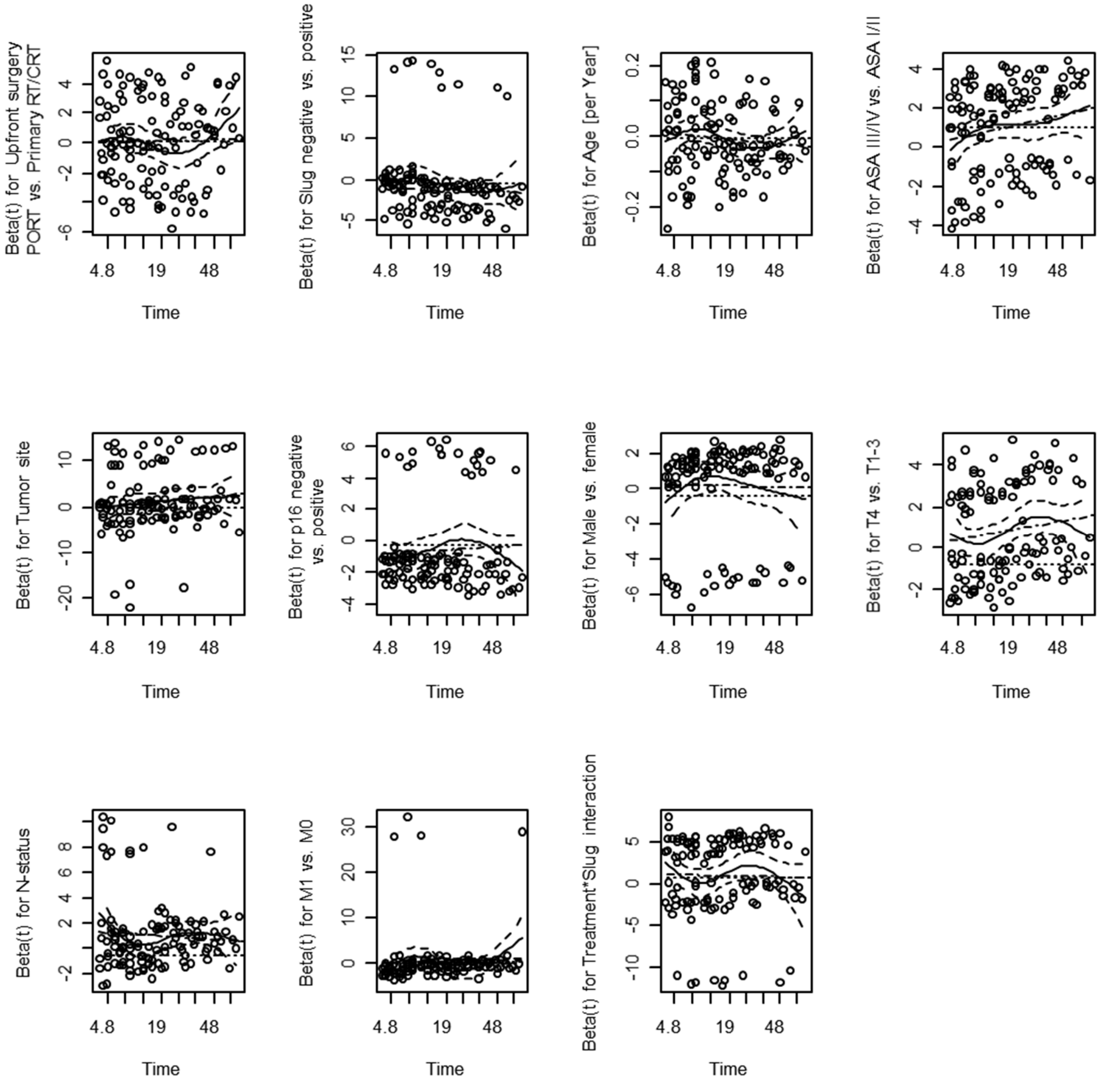

Figure S1. Scaled Schoenfeld residuals by time. Scaled Schoenfeld residuals obtained with the cox.zph function in the R Survival package (see main text). The regression lines of the scaled Schoenfeld residuals with survival time for uncensored cases did not substantially leave a horizontal 95% confidence band obtained with a loess smoother, indicating no relevant violation of the proportional hazard assumption.

Table S1. Details of treatment modalities.

First-Line Treatment Modality	Treatment Details	Count	Column %
Upfront surgery/PORT[1)]	Surgery only	79	48.5%
	Upfront surgery & postoperative radiotherapy	53	32.5%
	Upfront surgery & concurrent systemic therapy[2)] and RT[3)]	31	19.0%
	Subtotal	163	100.0%
Primary RT/CRT[4)]	Primary RT	21	12.8%
	Primary concurrent systemic therapy[2)] and RT[3)]	143	87.2%
	Subtotal	164	100.0%
Total		**327**	

(1) PORT: postoperative radiotherapy (RT) or postoperative systemic therapy/radiotherapy (CRT); (2) Systemic therapy includes chemotherapy and/or immunotherapy; (3) RT: radiotherapy; (4) CRT: Chemoradiotherapy including immunoradiotherapy and systemic therapy/radiotherapy.

Table S2. Characteristics of patients treated with upfront surgery PORT vs. primary RT/CRT.

		First-line Treatment Modality					
		Upfront Surgery/PORT		Primary RT/CRT		Total	
		Count	Column %	Count	Column %	Count	Column %
Gender	male	125	77%	136	83%	261	80%
	female	38	23%	28	17%	66	20%
ASA score	ASA I/II	102	63%	90	55%	192	59%
	ASA III/IV	61	37%	74	45%	135	41%
Smoking	< 10 pack-years	55	34%	62	38%	117	36%
	>= 10 pack-years	108	66%	102	62%	210	64%
Tumor site**	Lips and oral cavity	35	21%	13	8%	48	15%
	Oropharynx	57	35%	87	53%	144	44%
	Hypopharynx	20	12%	25	15%	45	14%
	Larynx	44	27%	30	18%	74	23%
	Others	7	4%	9	5%	16	5%
T stage (I–III vs. IV)***	T1–3	136	83%	100	61%	236	72%
	T4	27	17%	64	39%	91	28%
N stage***	N0	80	49%	29	18%	109	33%
	N1	34	21%	35	21%	69	21%
	N2	47	29%	85	52%	132	40%
	N3	2	1%	15	9%	17	5%
Distant metastasis*	M0	162	99%	156	95%	318	97%
	M1	1	1%	8	5%	9	3%
p16 status*	negative (≤60%)	130	80%	111	68%	241	74%
	positive (>60%)	33	20%	53	32%	86	26%
Slug IHC	negative	119	73%	123	75%	242	74%
	positive	44	27%	41	25%	85	26%

* $p \leq 0.05$; ** $p \leq 0.01$; *** $p < 0.001$.

Table S3. Results Cox proportional hazards analysis.

Covariate	Relative Risk[1)]	*p*-Value
Primary RT/CRT vs. upfront surgery PORT	1.08 (0.65 to 1.81)	0.759
Slug negative vs. positive	0.55 (0.25 to 1.19)	0.13
Age [per year]	1 (0.98 to 1.02)	0.67
ASA III/IV vs. ASA I/II	2.71 (1.76 to 4.16)	<0.001
Larynx vs. hypopharynx	0.73 (0.38 to 1.41)	0.35
Oral cavity vs. hypopharynx	0.76 (0.37 to 1.59)	0.472
Oropharynx vs. hypopharynx	0.7 (0.4 to 1.2)	0.193
Other sites vs. hypopharynx	0.47 (0.15 to 1.46)	0.191
p16 positive vs. negative	0.55 (0.33 to 0.93)	0.026
Male vs. female	1.16 (0.7 to 1.91)	0.572
T4 vs T1-3	2.19 (1.43 to 3.36)	<0.001
N1 vs. N0	1.13 (0.57 to 2.23)	0.73
N2 vs. N0	1.33 (0.8 to 2.21)	0.277
N3 vs. N0	4.85 (2.13 to 11.05)	<0.001
M1 vs. M0	1.24 (0.44 to 3.49)	0.689
Interaction of Slug IHC status * first-line treatment modality[2)]	3.09 (1.23 to 7.78)	0.017

[(1)] In brackets: lower and upper 95% confidence interval of relative risk; [(2)] Main outcome parameter.

Table S4. Proportional hazard tests. Proportional hazards assumption was tested with the cox.zph function from the R Survival package (see main text). The global test of proportional hazard assumptions was not significant and neither were the score tests for the single covariates, suggesting that the proportional hazard assumption of the model held.

Covariate	Chisq	df	*p*
Upfront surgery PORT vs. PrimaryRT/CRT	0.016	1	0.9
Slug negative vs. positive	21.96	1	0.142
Age [per year]	0.14	1	0.708
ASA III/IV vs. ASA I/II	24.31	1	0.106
Tumor site	53.79	4	0.254
p16 negative vs positive	0.51	1	0.481
Male vs. female	0.00004	1	1.032
T4 vs. T1-3	38.45	1	0.053
N-status	40.63	3	0.253
M1 vs. M0	0.33	1	0.557
Treatment*Slug interaction	14.54	1	0.233
GLOBAL	218.19	16	0.108

Table S5. Total patient population and patients with available Slug IHC status (study population). Between January 2008 and December 2019, 1124 inpatients with incident HNC were treated. Slug immunohistochemistry (IHC) status was available for 354 patients, which were included as study population.

		with Slug IHC		without Slug IHC		Total	
		Count	Column %	Count	Column %	Count	Column %
Gender	male	286	80.8%	597	77.5%	883	78.6%
	female	68	19.2%	173	22.5%	241	21.4%
	Total	354	100.0%	770	100.0%	1124	100.0%
Age at first diagnosis **	≤50	33	9.3%	105	13.6%	138	12.3%
	51–60	122	34.5%	216	28.1%	338	30.1%
	61–70	115	32.5%	235	30.5%	350	31.1%
	71–80	70	19.8%	151	19.6%	221	19.7%
	>80	14	4.0%	63	8.2%	77	6.9%
	Total	354	100.0%	770	100.0%	1124	100.0%
ASA score	ASA I/II	198	56.3%	239	58.9%	437	57.7%
	ASA III/IV	154	43.8%	167	41.1%	321	42.3%
	Total	352	100.0%	406	100.0%	758	100.0%
Smoking***	<10 pack-years	121	34.2%	190	46.5%	311	40.8%
	≥10 pack-years	233	65.8%	219	53.5%	452	59.2%
	Total	354	100.0%	409	100.0%	763	100.0%
Tumor site***	Lips/oral cavity	51	14.4%	131	17.0%	182	16.2%
	Oropharynx	158	44.6%	191	24.8%	349	31.0%
	Hypopharynx	48	13.6%	47	6.1%	95	8.5%
	Larynx	79	22.3%	182	23.6%	261	23.2%
	Others	18	5.1%	219	28.4%	237	21.1%
	Total	354	100.0%	770	100.0%	1124	100.0%
T stage (I–III vs. IV)**	T1–3	246	69.5%	593	77.2%	839	74.8%
	T4	108	30.5%	175	22.8%	283	25.2%
	Total	354	100.0%	768	100.0%	1122	100.0%
N stage***	N0	112	31.6%	370	48.2%	482	43.0%
	N1	72	20.3%	100	13.0%	172	15.3%
	N2	144	40.7%	272	35.4%	416	37.1%
	N3	26	7.3%	26	3.4%	52	4.6%
	Total	354	100.0%	768	100.0%	1122	100.0%
Distant metastasis*	M0	336	94.9%	749	97.3%	1085	96.5%
	M1	18	5.1%	21	2.7%	39	3.5%
	Total	354	100.0%	770	100.0%	1124	100.0%
p16 status	negative	262	74.2%	246	79.1%	508	76.5%
	positive	91	25.8%	65	20.9%	156	23.5%
	Total	353	100.0%	311	100.0%	664	100.0%
First line treatment modality***	Upfront surgery/PORT	163	49.8%	473	66.7%	636	61.4%
	PrimaryRT/CRT	164	50.2%	236	33.3%	400	38.6%
	Total	327	100.0%	709	100.0%	1036	100.0%

* $p \leq 0.05$; ** $p \leq 0.01$; *** $p < 0.001$.
